# Cost-effectiveness of pioglitazone in type 2 diabetes patients with a history of macrovascular disease: a German perspective

**DOI:** 10.1186/1478-7547-7-9

**Published:** 2009-05-05

**Authors:** Werner A Scherbaum, Gordon Goodall, Katrina M Erny-Albrecht, Massimo Massi-Benedetti, Erland Erdmann, William J Valentine

**Affiliations:** 1 Universität Düsseldorf, Klinik für Endokrinologie, Diabetologie und Rheumatologie; 2 IMS Health, Basel, Switzerland; 3 University of Perugia, Medicine and Metabolic Diseases, Perugia, Italy; 4 Medizinische Klinik III der Universität zu Köln, Köln, Germany

## Abstract

**Background:**

The aim of this study was to project health-economic outcomes relevant to the German setting for the addition of pioglitazone to existing treatment regimens in patients with type 2 diabetes, evidence of macrovascular disease and at high risk of cardiovascular events.

**Methods:**

Event rates corresponding to macrovascular outcomes from the Prospective Pioglitazone Clinical Trial in Macrovascular Events (PROactive) study of pioglitazone were used with a modified version of the CORE Diabetes Model to simulate outcomes over a 35-year time horizon. Direct medical costs were accounted from a healthcare payer perspective in year 2005 values. Germany specific costs were applied for patient treatment, hospitalization and management. Both costs and clinical benefits were discounted at 5.0% *per annum*.

**Results:**

Over patient lifetimes pioglitazone treatment improved undiscounted life expectancy by 0.406 years and improved quality-adjusted life expectancy by 0.120 quality-adjusted life years (QALYs) compared to placebo. Direct medical costs (treatment plus complication costs) were marginally higher for pioglitazone treatment and calculation of the incremental cost-effectiveness ratio (ICER) produced a value of €13,294 per QALY gained with the pioglitazone regimen versus placebo. Acceptability curve analysis showed that there was a 78.2% likelihood that pioglitazone would be considered cost-effective in Germany, using a "good value for money" threshold of €50,000 per QALY gained. Sensitivity analyses showed that the results were most sensitive to changes in the simulation time horizon. After adjustment for the potential stabilization of pancreatic β-cell function with pioglitazone treatment, the ICER was €6,667 per QALY gained for pioglitazone versus placebo.

**Conclusion:**

The findings of this modelling analysis indicated that, for patients with a history of macrovascular disease, addition of pioglitazone to existing therapy reduces the long-term cumulative incidence of diabetes-complications at a cost that would be considered to represent good value for money in the German setting.

## Introduction

The direct cost of care for patients with diabetes accounts for 14.2% of total health care costs in Germany, and as the number of diagnosed type 2 diabetes patients continues to rise this is likely to increase substantially in the future [1]. However the cost of diabetes in Germany is not evenly distributed, with approximately 15% of patients being responsible for almost 60% of all direct costs and the presence of diabetes-related complications being the most important driver of increasing costs [1,2]. Targeting of resources to these cost-intensive patients with, or at high risk for, complications may represent a more pragmatic and effective strategy on which to base healthcare policy aimed at containing the current escalation in diabetes-related costs in Germany [1,3].

Based on data relating to 809 patients, the German arm of the Cost of Diabetes in Europe-type 2 (CODE-2) conducted in 1998 identified complications as the greatest contributor to direct costs of diabetes care [4]. In CODE-2, relative to no complications the presence of either microvascular or macrovascular complications increased direct costs by two-fold whilst for patients with both microvascular and macrovascular complications costs were increased by four-fold. Similarly in the more recent German Cost of Diabetes Mellitus (CoDiM) study of 26,971 diabetes patients insured by a large health insurance fund (AOK-Hessen) between 1998 and 2002, the mean annual cost per patient with at least one complication was 2.5-fold higher compared to those without complications (€6,766 versus €2,756) [3]. Corresponding values for patients with two or three complications versus those without complications were a 2.9 fold (€8,077 versus €2,756) and 4.7 fold (€12,939 versus €2,756) increase, respectively [3]. Epidemiological surveys of representative patient groups in Germany have shown that many diabetes patients fail to achieve adequate glycaemic control (HbA1c = 6.5%) and almost half of all patients have at least one diabetes-related complication [1,3,5-7].

In the CoDiM study, 41% of the 11,983 patient treated with oral antidiabetic (OAD) agents alone reportedly had macrovascular disease, and the corresponding values were 52% and 44% for patients treated with OAD plus insulin or diet alone respectively [3]. In the earlier CODE-2 study 50% of the type 2 diabetes patients had at least one complication [1]. More recently the Diabetes Cardiovascular Risk Evaluation: Targets and Essential Data for Commitment of Treatment (DETECT) Germany-wide study conducted in 2003 and including 8,188 type 2 diabetes patients reported that 50% of patients had at least one diabetes complication and 34% had macrovascular complications [6]. Therefore, there exists a clear need from both a clinical and economic view for more effective intervention among these patients at increased risk for further complications and for progressive worsening of established complications.

The thiazolidinedione (TZD) class of anti-hyperglycaemic agents act via stimulation of the peroxisome proliferator activated receptor-γ (PPAR-γ) to increase sensitivity to insulin in muscle, liver and adipose tissue and thereby improve glycaemic control [8,9]. The TZD, pioglitazone, has been shown in randomized clinical trials to significantly lower blood glucose and to improve lipid abnormalities in patients with type 2 diabetes when used as monotherapy or in combination with other oral antihyperglycaemic agents or insulin [10-13]. In addition to this, and in contrast to metformin and sulphonylureas (SU), recent studies have provided evidence of pioglitazone associated improvement in pancreatic β-cell function that correlates strongly with improved glycaemic control in patients with type 2 diabetes [14]. The European Medicines Agency (EMEA) has recently extended the approved indications for pioglitazone to include use in combination with insulin for patients with insufficient control on insulin and a contraindication or intolerance for metformin. Pioglitazone is also indicated for use as monotherapy in patients inadequately controlled with diet and exercise and for whom metformin is contraindicated; as dual oral therapy in combination with metformin or SU for patients with inadequate control and SU or metformin intolerance respectively; as triple oral therapy in combination with metformin and a SU for patients failing to achieve adequate control with dual oral therapy [15].

Of particular relevance to the needs of type 2 diabetes patients in Germany, the PROspective pioglitAzone Clinical Trial in macroVascular Events (PROactive) investigated prospectively the effect of pioglitazone, used as an "add on" to normal treatment regimens, on macrovascular outcomes in patients with a history of macrovascular events and demonstrated that treatment was associated with a reduced incidence of cardiovascular events [16,17]. The PROactive study was conducted in 321 centres in 19 European countries and recruited 5,238 type 2 diabetes patients who were followed up for an average of 34 months. At the end of study, there was a 10% relative risk reduction with pioglitazone in the primary endpoint (p = 0.09, non-significant), which was a composite of all cause mortality, non-fatal myocardial infarction (MI) (including silent MI), stroke, acute coronary syndrome (ACS), endovascular or surgical intervention in the coronary or leg arteries, and amputation above the ankle. For the principal secondary composite endpoint (all cause mortality, MI or stroke) treatment with pioglitazone was associated with a significant reduction compared to placebo (hazard ratio = 0.84, p = 0.027). By the end of PROactive, patients receiving pioglitazone reported significant improvements in HbA1c, high-density lipoprotein (HDL) cholesterol and triglycerides compared to those receiving placebo [17]. In the PROactive study, pioglitazone reduced the number of patients progressing to long-term insulin therapy by half. In addition to this, evidence supporting the potential of TZDs to stabilize pancreatic function and hence glycaemic control continues to accumulate, with a number of reports based on clinical observations in type 2 diabetes patients now supporting earlier evidence gained in animal models [14,18].

These findings demonstrate that improved glycaemic and lipid control associated with pioglitazone treatment lead to a reduced incidence of macrovascular events and in contrast to most currently available treatment options use of pioglitazone could potentially delay the progressive decline of pancreatic function that undermines attempts to achieve adequate glycaemic control. In view of the substantial burden imposed by diabetes-related complications on German healthcare resources, uptake of pioglitazone by patients at high risk for complications or progression of existing complications may represent an important treatment option in both clinical and economic terms. However as increasing demands are being placed on limited healthcare resources there is a need to demonstrate that treatments are both effective in clinical terms and affordable in economic terms if they are to be made available to those most likely to benefit. The objective of this study was to estimate the impact of pioglitazone treatment on life expectancy, quality-adjusted life expectancy and incidence of macrovascular events and to account for direct medical costs over a patient lifetime horizon in the German setting.

## Methods

### Model

Short-term clinical effects of the pioglitazone and placebo treatment regimens from PROactive were used to project long-term outcomes using a modified version of the extensively published and validated CORE Diabetes Model [19,20].

The CORE Diabetes Model is a computer simulation model developed to determine the long-term health outcomes and economic consequences of interventions in diabetes. Disease progression is modelled via 15 inter-dependent semi-Markov sub-models that simulate progression of disease related complications (angina, MI, congestive heart failure, stroke, peripheral vascular disease, diabetic retinopathy, macula oedema, cataract, hypoglycaemia, ketoacidosis, lactic acidosis, nephropathy and end-stage renal disease, neuropathy, foot ulcer, amputation and non-specific mortality). Each sub-model uses time, state and diabetes type-dependent probabilities derived from published sources. Using the CORE Diabetes Model diabetes management strategies can be compared in different patient populations in a variety of realistic clinical and economic settings.

In the current study, short-term (0 – 3 years) data from PROactive was used as a basis for long-term projections using the CORE Diabetes Model adapted to include clinical input data from PROactive [21]. For a more detailed account of the PROactive long-term simulation model describing the adaptation of the original CORE Diabetes Model, the transition probabilities applied and the utility values accounted for disease states interested readers are encouraged to view the publication of Valentine *et al*. [21].

**Figure 1:**
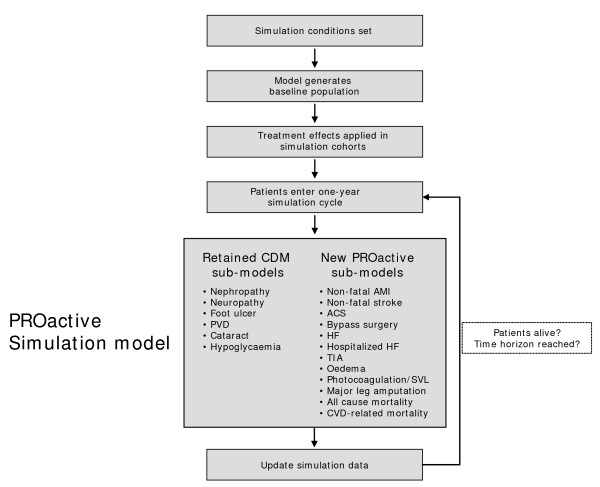
**Overview of the PROactive long-term simulation model**.

In brief, to incorporate the PROactive clinical and adverse event endpoint data into the CORE Diabetes Model, a number of new complication sub-models were developed or modified. The overall architecture of the modified CORE Diabetes Model remained analogous to the original version, with all active sub-models running in parallel to simulate the progression of disease and the development of diabetes-related complications. The sub-models included in the final PROactive long-term simulation model were; acute coronary syndrome, percutaneous coronary intervention (PCI), coronary artery bypass graft (CABG), bypass surgery/revascularization of the leg, hospital admission for heart failure, non-serious heart failure, oedema, myocardial infarction, transient ischemic attack (TIA), stroke, photocoagulation, severe vision loss, nephropathy, peripheral vascular disease, diabetic foot, amputation, and cataract (Figure 1).

Event rates in the placebo arm were calculated directly from the annual hazard rates observed over months 0–36 of PROactive assuming constant risk [21]. To calculate event rates in the pioglitazone arm hazard ratios were applied to the event rates from the placebo arm in line with the relative risk observed for each event during PROactive. To capture the statistical uncertainty in the trial data, hazard ratios in the modelling simulations were randomly sampled from a lognormal distribution generated from μ and σ values derived directly from the trial data. Constant risk was assumed for application of hazard ratios to calculate event rates in the pioglitazone arm for all endpoints with the exception of oedema, where hazard ratios were sampled from separate distributions for the first and subsequent years of the simulation to capture the decrease in risk after the first year of treatment with pioglitazone (see Additional file 1).

All-cause and cardiovascular mortality rates were derived directly from the PROactive Study for years 1–3 of the simulation, with rates subsequently doubling every 10 years [22]. Event rates for following years were calculated by applying relative risk adjustments from the United Kingdom Prospective Diabetes Study (UKPDS) and Framingham data for each additional life-year gained (*i.e*. as the patient gets older, his/her risk of experiencing an event increases) [23-27]. In all sub-models, the occurrence of events resulted in the accounting of event costs and, where applicable, subsequent state costs as well as assignment of the appropriate disutility values.

The modified CORE Diabetes Model estimated the impact of treatment on life expectancy, quality-adjusted life expectancy (based on CODE-2 utilities), diabetes-related complications (cumulative event rates), direct medical costs and cost-effectiveness ratios over patient lifetimes, in line with specifications for health economic evaluations and under the assumption that treatment allocation continues [28,29].

### Simulation Cohorts

A simulation cohort of patients was defined with baseline demographics, and complications representative of the two treatment arms from PROactive. Long-term outcomes were calculated 1,000 times in the model using a simulated population of 1,000 patients, in order to capture the effects of random variation between individual patients. At baseline the simulation cohort had a mean age of 61.8 years, duration of diabetes 10 years, and a mean HbA1c level of 8.1% (Table 1). For the purposes of this analysis patients were assumed to remain on the same treatment regimen for the duration of the simulation (35 years or death).

**Table 1:** Baseline characteristics of the simulation cohort

**Characteristic**	**Value**	**SD**	**Data Source**
**Demographics**			
Proportion male (%)	66.1	--	PROactive
Mean age (years)	61.8	7.7	PROactive
Duration of diabetes (years)	10	7	PROactive
Ethnic Group			
Proportion White (%)	98.6	--	PROactive
Proportion Black (%)	1.4	--	PROactive
**Baseline risk factors**			
HbA1c (%-points)	8.1	1.4	PROactive
Systolic blood pressure (mmHg)	143.4	17.8	PROactive
BMI (kg.m^-2^)	30.9	4.8	PROactive
HDL-C (mmol/l)	1.2	0.3	PROactive
LDL-C (mmol/l)	3.0	1.0	PROactive
Total cholesterol (mmol/l)	2.3	0.9	PROactive
Triglycerides (mmol/l)	2.2	1.8	PROactive
Proportion smokers (%)	13.8	--	PROactive
**Baseline complications**			
ACS (%)	13.65	--	PROactive
CABG/PCI (%)	30.75	--	PROactive
PVD (%)	24.3	--	PROactive
MI (%)	47.0	--	PROactive
Stroke (%)	19.0	--	PROactive
Microalbuminuria (%)	14.3	--	PROactive
Neuropathy (%)	25.6	--	PROactive

HbA1c = glycosylated haemoglobin; BMI = body mass index; HDL-C = high density lipoprotein cholesterol; LDL-C = low density lipoprotein cholesterol; ACS = acute coronary syndrome; CABG = coronary artery bypass graft; PCI = percutaneous coronary intervention; MI = myocardial infarction

The use of angiotensin converting enzyme inhibitors (ACE inhibitors) has an important influence on renal outcomes and in the simulation cohort this was set to 62.8% based on data from PROactive. Use of aspirin and statin medication has an impact on cardiovascular events, and in line with the PROactive study cohort 73.1% and 42.9% of the simulation cohort were assigned to receive these treatments respectively. Therefore, risk adjustment for the use of aspirins or statins was disabled in the analysis as the influence of these agents was already taken into account, along with the impact of ACE inhibitors, in the cardiovascular event rates taken from PROactive. It is relevant to note here that a survey of prescription drug costs for diabetic patients in Germany reported that approximately 45% of patients with type 2 diabetes were treated with ACE inhibitors and 25% with statins in 2004 [30]. Additional settings for patient management parameters (e.g. screening for renal disease) were set in line with the standard of care patients in the PROactive study were receiving, with all patients receiving regular screening and foot check-ups.

### Treatment effects

For the base case simulation, the clinical effects associated with the pioglitazone and placebo treatment regimens were applied as observed during PROactive. Treatment effects on HbA1c were applied separately in simulation years 1, 2 and 3, and in subsequent years, based on the findings from PROactive (for long-term projection). Changes in HbA1c and other parameters for pioglitazone and placebo regimens were applied as summarized in Table 2 and based on the statistical report compiled by Nottingham Clinical Research Limited for the PROactive study group. After year three of PROactive the between treatment group difference in HbA1c was -0.5% for pioglitazone versus placebo. The long-term progression of all of these clinical parameters subsequently followed the patterns previously described by Palmer *et al. *in their description of the CORE Diabetes Model [19].

**Table 2:** Summary of base case intervention effects

**Effect**	**Mean change from baseline**	
	**Pioglitazone**	**Placebo**
Change in HbA1c in year 1 (%-points)	-0.9	-0.3
Change in HbA1c in year 2 (%-points)	+0.1	+0.1
Change in HbA1c in year 3 (%-points)	+0.3	+0.2
Change in subsequent years	+0.15	+0.15
Total cholesterol (mmol/l)	+0.39	+0.25
HDL-C (mmol/l)	+0.54	+0.30
LDL-C (mmol/l)	+0.35	+0.22
Triglycerides (mmol/l)	-0.064	+0.076
Systolic blood pressure (mmHg)	-3.8	-2.4
BMI (kg.m^-2^)	+1.1	-0.1
Overall hypoglycaemic event rate (per 100 patient years)*	+9.29	+6.68

HDL-C = high density lipoprotein cholesterol; LDL-C = low density lipoprotein cholesterol; BMI = body mass index.

* Includes both minor events not requiring assistance [726 patients on pioglitazone and 528 on placebo, (p < 0.0001)] and major events that resulted in admission to hospital [19 patients on pioglitazone and 11 patients on placebo (p = 0.14)].

### Costs

Direct medical costs were expressed in 2005 Euro (€) and taken from Germany specific sources where possible (Table 3). Unit costs retrieved from published sources that were not expressed in 2005 € values were inflated using indices from the German Federal Statistics Office . Direct medical costs were calculated as the sum of treatment costs (based on data from PROactive), patient management costs and the cost of complications. The annual costs of study medication were accounted based on data from PROactive and a mean annual cost of €736.15 per patient for pioglitazone and zero for placebo. Calculations were based on daily costs of €1.29, €1.93 and €2.31 for treatment with 15 mg, 30 mg and 45 mg pioglitazone and zero cost for placebo.

**Table 3:** Cost per event or state used in the analysis, expressed in 2005 values (€)

	**Event cost (€)**	**Follow up cost (€)**	**Reference(s)**
Death (all causes)	0.00	0.00	Assumed
CVD death	0.00	0.00	Assumed to be zero as no published value was found
MI (excluding silent MI)	8,634.93	3,647,32	[49]
Silent MI	0.00	0.00	Assumed
Acute coronary syndrome (ACS)	3,120.92	0.00	[50]
CABG only	12,435.81	0.00	[51]
PCI only	4,360.03	0.00	[51]
Stroke	10,523.62	6,178.06	[49] and [52]
Leg amputation (major, above ankle)	15,405.10	3,303.92	[49] and [53]
Bypass surgery/revascularization of leg	6,268.09	0.00	[50]
Transient ischemic attack (TIA)	2,353.52	0.00	[50]
Retinal photocoagulation	1,862.28	340.33	[50]
Severe vision loss (SVL)	10,660.98	10,660.98	[54]
Hospitalization for CHF	2,272.97	0.00	[55]
Non-serious heart failure	34.51	0.00	Assumed to be same as a physician visit cost (as CVD medication costs are already captured)
Oedema	34.51	0.00	[56]
Peripheral vascular disease (onset)	2,635.85	0.00	[56]
Haemodialysis	59,248.85	59,248.85	[57]
Peritoneal dialysis	47,198.66	47,198.66	[57]
Kidney transplant	69,504.00	11,116.77	[58]
Cataract extraction	1,347.78	0.00	[59]
Neuropathy, onset	3,930.36	0.00	[60]
Uninfected ulcer	894.22	0.00	[60]
Infected ulcer	1,818.58	0.00	[60]
Gangrene	3,247.62	0.00	[60]
Major hypoglycaemic event	2,555	0.00	[34]

CVD = cardiovascular disease; MI = myocardial infarction; CABG = coronary artery bypass graft; PCI = percutaneous coronary intervention; CHF = congestive heart failure. Where necessary costs were inflated to 2005 values

### Health state utilities

For the base case analysis, health state utilities for the events reported in PROactive were derived wherever possible from the CODE-2 study [31]. Where PROactive events were not taken into consideration in the CODE-2 formula, no substitute values were used (as this could have produced erroneous findings). All other quality of life utilities used in the base case simulation have been described previously by Palmer *et al*. [19]. Sensitivity analyses were performed to investigate the impact of including additional quality of life disutilities on the base case findings. A list of all PROactive model specific utilities incorporated into the model is given in Additional file 2.

### Discounting, Time Horizon and Perspective

Discounting was applied to costs, life expectancy and quality-adjusted life expectancy at an annual rate of 5.0% in line with current recommendations for the German setting [32]. To capture all relevant long-term complications, a lifetime horizon of 35 years was used in the analysis. The analysis was conducted from a third-party healthcare payer perspective in Germany.

### Sensitivity Analyses

Sensitivity analyses were performed to investigate the impact of key input variables on the projected outcomes of the analysis. Typically these were univariate sensitivity analyses where the base case parameters were left unchanged except for the parameters specifically called out in the descriptions here. In the base case, changes in HbA1c were simulated using data from PROactive in the first three years and thereafter (years 4+) an annual increase in HbA1c of 0.15% was assumed in line with observations from UKPDS [33]. To investigate the impact of potential benefits with respect to β-cell function a sensitivity analysis was performed where the annual HbA1c creep of 0.15% was reduced to 0.1% for years 4+. There were very few hypoglycaemic events observed in the PROactive trial but to investigate the potential impact of these on the overall outcomes a sensitivity analysis was performed where all events were associated with a cost of €2,555 in line with data from Holstein *et al*. [34]. The impact of time horizon was investigated by varying this between 10 and 35 years. Similarly, the impact of discount rates was assessed by varying this between zero and 10% in line with recommendations of the Hanover consensus [29]. Treatment related assumptions were investigated by varying the impact of pioglitazone treatment on HbA1c such that both pioglitazone and placebo arms simulated a change in HbA1c of -0.3% corresponding to the PROactive placebo arm. The influence of risk adjustment for age on the event rates taken from PROactive was investigated by performing an analysis where no risk adjustment for age was applied during the simulation (risk adjustment factors all set to 1). In order to examine the effect of uncertainty on costs accounted in the model, sensitivity analyses were conducted varying the unit cost of pioglitazone treatment by +/- 20% and then also by varying all other costs (complication and management) by the same margins.

In the base case scenario quality of life utilities were taken from CODE-2, and for many events such as oedema and heart failure this resulted in no disutilities being applied due to the absence of data. To investigate the influence of including quality of life disutilities not included in the CODE-2 formula a number of sensitivity analyses were run to include additional quality of life disutilities as previously described [21]. In brief, this involved repeating the base case analysis using the CORE default method of quality-adjusted life expectancy estimation, applying an event disutility of -0.01 for oedema and a follow up disutility of zero (as the condition is typically short-lived), applying an event disutility to hospitalization for heart failure of -0.121 and a follow up disutility of -0.181, based on the UKPDS [35], or applying an event disutility of -0.0605 for non-serious heart failure and a follow up disutility of zero (due to the absence of published data). Utility values for oedema and non-serious heart failure were based on assumptions.

A "worst case" scenario was also included in the sensitivity analysis where event disutilities for oedema (as described), hospitalization for heart failure (by applying an event disutility of -0.121 and a follow up disutility of -0.181) and revascularization of the leg (by applying an event disutility of -0.059 and a follow up disutility of zero, assuming a disutility comparable to that reported for CABG) from the post-hoc study of Lescol Intervention Prevention Study were applied [36].

### Statistical Methodology

The health economic analysis was performed using a non-parametric bootstrapping approach in which the progression of diabetes was simulated in 1,000 patients through the model 1,000 times to calculate the mean and standard deviation of costs, life expectancy and quality-adjusted life expectancy using second order Monte Carlo simulation [37]. Mean results from each of the 1,000 iterations were used to create a scatter plot, comparing the differences in costs and outcomes for pioglitazone and placebo treatment regimens. These values were in turn used to generate a cost-effectiveness acceptability curve over a range of willingness to pay values in the German setting.

## Results

### Life expectancy and quality-adjusted life expectancy

The current health-economic analysis indicated that based on clinical findings for PROactive and long-term projections with a modified version of the CORE Diabetes Model, treatment with pioglitazone was associated with improvements in life expectancy and quality-adjusted life expectancy compared to placebo. Mean life expectancy (discounted by 5.0% *per annum*) increased by 0.172 years with pioglitazone and after adjustment for quality of life an improvement of 0.120 quality-adjusted life years (QALYs) was projected versus placebo (Table 4). When no discounting was applied to the long-term outcomes, mean life expectancy in the pioglitazone treatment arm was 0.406 years longer than in the placebo arm.

**Table 4:** Summary of base case results for pioglitazone versus placebo

**Outcome (all reported values are discounted)**	**Pioglitazone**	**Placebo**	**Difference (PIO – PLA)**
**Clinical outcomes**			
			
Life expectancy (years)	10.044 (0.140)	9.871 (0.139)	0.172
			
Quality-adjusted life expectancy (QALYs)	7.543 (0.102)	7.422 (0.102)	0.120
			
**Cost outcomes**			
			
Total direct costs (€)	105,433 (2,650)	103,834 (2,618)	1,599
			
Incremental cost-effectiveness based on life expectancy	€9,281 per life year gained		
			
Incremental cost-effectiveness based on quality-adjusted life expectancy	€13,294 per QALY gained		

Values shown are means with standard deviation in parentheses; PIO = pioglitazone; PLA = placebo; QALY = quality-adjusted life-years. Incremental values are given as the pioglitazone value minus the placebo value.

### Lifetime costs and cost-effectiveness

Over patient lifetimes, treatment with pioglitazone was associated with higher direct medical costs than the placebo regimen (Table 4). Direct costs increased by €1,599 with pioglitazone compared to placebo. This increase was largely due to increased treatment costs (€27,210 versus €24,348), whilst complication-related costs decreased by €1,263 (Table 5). Treatment with pioglitazone was associated with a reduced cost for stroke events by €1,487 and an increase in costs by €274 for hospitalization for heart failure compared to placebo (although mortality rates from heart failure did not differ between groups).

**Table 5:** Breakdown of direct costs for pioglitazone versus placebo over a lifetime horizon

**Cost (per patient)**	**Pioglitazone (€)**	**Placebo (€)**	**Difference (PIO – PLA) (€)**
Treatment	22,941	20,151	2,790
Management	4,269	4,197	72
Total complication costs	78,223	79,486	-1,263

Total direct costs	105,433	103,834	1,599

Total direct medical costs were estimated as the sum of pharmacy costs based on resource use from PROactive, patient management costs (screening for retinopathy, nephropathy and foot ulcers), and the costs of all diabetes-related complications captured in the analysis. Values shown are means.

**Figure 2:**
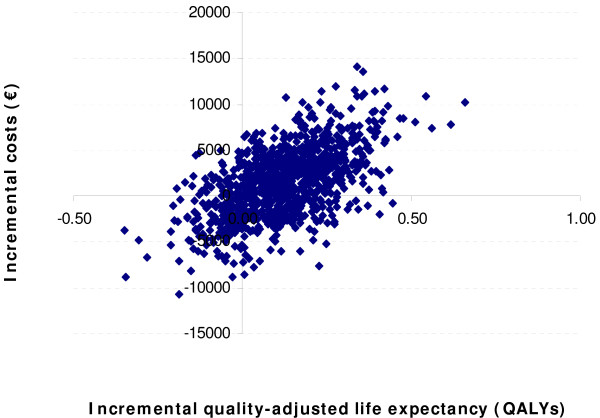
**Cost-effectiveness scatter plot of incremental costs and incremental effectiveness for pioglitazone versus placebo**.

**Figure 3:**
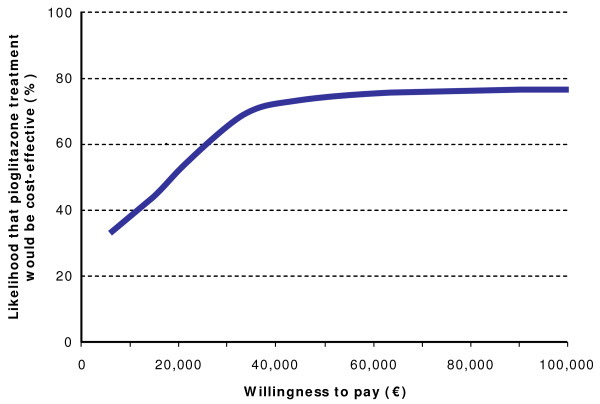
**Cost-effectiveness acceptability curve for pioglitazone versus placebo**.

Estimation of incremental cost-effectiveness ratios (ICER) for pioglitazone versus placebo treatment produced values of €9,281 per life year gained and, taking quality of life into account, €13,294 per QALY gained (Table 4). The values from the 1,000 means (each from 1,000 patients) of incremental costs and incremental effectiveness (in terms of quality-adjusted life expectancy) were used to generate a scatter plot on the cost-effectiveness plane. This analysis shows that the majority of points were in the upper right quadrant of the plane, indicating increased effectiveness and increased costs associated with pioglitazone treatment over placebo. These values were then used to create a cost-effectiveness acceptability curve by assessing what proportion of values fell below set willingness to pay values (Figure 2). The analysis demonstrated that, with a willingness to pay of €50,000 per QALY in the German setting, there was a high probability (78.2%) that pioglitazone would be cost-effective (Figure 3).

### Sensitivity Analyses

Sensitivity analysis showed that the results were most sensitive to variation in the time horizon and assumptions on the duration of the benefits of pioglitazone treatment observed in the trial, whilst the exclusion of improved β-cell function from the base case scenario was shown to bias against pioglitazone (see Additional file 3).

To simulate a pioglitazone-associated delay in β-cell deterioration the annual HbA1c creep was reduced from 0.15% to 0.10%, over a 35-year time horizon the projected quality-adjusted life expectancy improved and direct costs were reduced such that pioglitazone became dominant compared to the base case value of €13,294 per QALY gained, this was primarily due to pioglitazone being less costly (€102,954 versus €103,834). In the scenario where the cost for any hypoglycaemic event was set at €2,555 the ICER increased slightly to €13,980 for pioglitazone versus placebo. Reducing the time horizon to ten years (compared to the base case of 35 years) increased the ICER compared to the base case. This occurs because shorter time horizons fail to capture the development of many end-stage complications, such as end-stage renal disease, severe vision loss and macrovascular complications, some of which would be avoided in the pioglitazone treatment group. A time horizon of 20 years was associated with a moderately improved ICER compared to the base case (€10,144 per QALY gained). This outcome is most likely due to the increased life expectancy of patients receiving pioglitazone compared to placebo, such that beyond 20 years there are more surviving patients and hence they accumulate greater costs.

Addition of various disutility values for inclusion in the analysis had very little effect on the relative findings of the analysis with ICERs remaining well below the willingness-to-pay threshold of €50,000 per QALY gained. Assuming no difference between treatments in HbA1c change also had very little impact on outcomes, and this was in line with the modest between treatment group difference in HbA1c from year 4+ and the cohort HbA1c of 8.1% at baseline. Nevertheless, the improved cardiovascular risk profile associated with pioglitazone meant that direct costs were reduced (€1,412 versus €1,599), which in turn meant that the ICER was lower than in the base case; €11,870 per QALY gained for pioglitazone versus placebo.

By reducing the unit cost of pioglitazone by 20%, from €736.15 to €588.92, resulted in a lower ICER of €424 per QALY gained for pioglitazone versus placebo. Furthermore increasing the cost of pioglitazone by 20% to €883.38 resulted in an ICER of €26,162 per QALY gained. When altering all other costs inputs in the model by +/- 20% the incremental costs and corresponding ICERs for pioglitazone versus placebo were €1,447 and €12,035 for a reduction in costs of 20% and €1,750 and €14,553 for an increase in costs of 20%, respectively.

## Discussion

Based on outcomes from PROactive the addition of pioglitazone to current treatment regimens would be associated with an ICER of €13,294 per QALY gained when viewed over a lifetime time horizon in the German setting. In the base case analysis, treatment with pioglitazone was associated with improvements in life expectancy of 0.172 years and quality-adjusted life expectancy of 0.120 QALYs, and slightly higher direct medical costs (€1,599) over patient lifetimes.

Results from both the German arm of the CODE-2 and the more recent German CoDiM study identified treatment of complications as the main driver of direct costs [1,3,4,38]. Meanwhile three very large epidemiological surveys conducted in 2001 and 2003 indicate that 33–50% of currently diagnosed type 2 diabetes patients in Germany have a history of macrovascular events and approximately 50% have inadequate glycaemic control [3,6]. Therefore the European cohort of patients with a history of macrovascular events included in PROactive and hence the outcomes reported from PROactive, are highly relevant to many type 2 diabetes patients currently cared for in the German setting. Treatment with pioglitazone lowers blood glucose and improves lipid abnormalities. For patients at high risk for macrovascular events, epidemiological studies such as the UKPDS have clearly demonstrated that multifactorial intervention to improve blood pressure and lipids as well as glycaemic control is likely to have a greater impact on the occurrence of future events than relying solely on improvement in HbA1c [39]. It is to be noted that the UKPDS recruited newly diagnosed patients as opposed to established patients.

In addition to providing evidence that improved glycaemic control reduces the incidence of complications, the UKPDS also demonstrated the progressive deterioration of glycaemic control despite use of antidiabetic medication. This occurs primarily as a consequence of declining pancreatic β-cell function, however most currently available treatment options for diabetes do not address this underlying cause of deteriorating control in diabetes. In contrast, use of the orally available pioglitazone is associated with improved glycaemic control via its insulin-sensitizing effects and has the potential to stabilize pancreatic function and hence stabilize glycaemic control [14,40]. Adjustment of the current analysis to take this into consideration further improved the cost-effectiveness of pioglitazone versus placebo, resulting in an ICER of €6,667 per QALY gained.

As noted in a recent review of the cost of diabetes in Germany by Liebl, the reporting of outcomes in terms of patient benefits including duration and quality of life are of particular importance in formulating healthcare policy [1]. Therefore it was important to perform appropriate sensitivity analyses to explore the robustness of projected outcomes to changes in the quality of life utilities assumed in the current analysis.

Although the quality of life utilities reported from CODE-2 were specific for European patients with type 2 diabetes the utilities were based on 4,641 study participants from Belgium, Italy, Netherlands, Spain and Sweden, and therefore a potential weakness of the current study is that these may not be representative of attitudes among German patients. The lack of Germany specific quality of life data was recently highlighted in a review of German economic evaluations of healthcare [41]. Until newer studies are conducted and published, the use of quality of life data taken from European patients with type 2 diabetes as utilized in the present investigation currently constitutes the best evidence available regarding likely preferences of German type 2 diabetes patients.

In addition to this a second potential weakness of the analysis presented here, in terms of quality of life, was the conservative approach taken to the estimation of quality-adjusted life expectancy using only data from CODE-2. This estimation did not capture changes in quality of life associated with several macrovascular endpoints (MI, ACS, PCI, CABG, TIA, oedema or revascularization of the leg). It is possible that this methodology may underestimate the improvements in quality-adjusted life expectancy as the formula does not capture some of the benefits of pioglitazone treatment (reduced rates of MI, ACS, PCI and CABG) or certain disadvantages such as oedema (although this was partially captured by the inclusion of body mass index [BMI] disutility data), hospitalization for heart failure and revascularization of the leg. This was addressed in the sensitivity analysis by including quality of life disutilities related to these endpoints and resulted in ICERs that were higher than projected in the base case (range €13,660–24,807 per QALY gained versus base case €13,294 per QALY gained). However it should be noted that this approach did not take into consideration potential improvements in quality of life resulting from decreased macrovascular events, and therefore is likely to bias against pioglitazone treatment.

As with all economic evaluations cost the accounting of costs is a potential area of weakness and should be adequately investigated for it's effect on outcomes. Sensitivity analyses have been performed to this end by varying cost inputs by +/- 20% and the resultant outcomes have been shown to be largely unchanged. It should also be noted that some cost inputs used in the analysis are ten years old and although this is not ideal, in the absence of any other data these are likely to provide better cost estimates than other commonly used options (e.g. anecdotal estimates from clinicians), even when inflated to current values using composite price indices.

Although the model bases outcomes primarily on the results of PROactive which was designed to report macrovascular endpoints, the model also captures other complications previously projected by the CORE Diabetes Model (nephropathy, neuropathy, peripheral vascular disease, foot ulcers and cataract). These sub-models use published data to account the risk of onset and progression and it should be acknowledged that these risks may differ for the patient population from PROactive. This uncertainty is unavoidable when constructing a non-trial based model but the errors are reduced somewhat by performing comparative analyses where inherent errors are applied to both interventions being compared.

Cost-effectiveness acceptability curve analysis indicated that there would be a 78.2% likelihood that pioglitazone would be cost-effective with a willingness to pay threshold of €50,000 per QALY gained. Although this threshold is frequently cited in health economic evaluations, very few countries or healthcare authorities have set an explicit threshold [42]. One exception to this is the Dutch Council for Public Health and Health Care who, in 2006, advised the government to set an explicit limit of €80,000 per QALY gained for diseases or injuries with a burden of 1.0 (causing a complete loss of all otherwise remaining QALYs) when making reimbursement decisions [43]. Further reporting of these recommendations indicated that for a burden of 0.5 the maximum threshold would be €40,000 under the recommendations of the Dutch Council [44]. Although a threshold range of £20,000–£30,000 per QALY gained has frequently been cited for the UK setting an explicit threshold has not been defined by the National Institute for Health and Clinical Excellence (NICE) [45-47]. In their detailed analysis of 33 economic evaluations previously brought before NICE, Devlin and Parkin noted an upper threshold of £47,000–£50,000 per life year gained for the UK setting [45]. In view of these apparent or likely thresholds for other European countries the threshold of €50,000 would seem to represent a reasonable assumption in the absence of explicit thresholds for Germany.

A recent commentary on the implications of PROactive in clinical practice showed that for treatment with pioglitazone the number needed to treat (NNT) to prevent either the combined primary or combined secondary cardiovascular endpoint (NNT 50 and 49 respectively) was well within the range reported for studies investigating the use of statins or ACE inhibitors for the prevention of cardiovascular events [48]. The current investigation has shown that based on use of a model that specifically incorporates outcome data (both for hard endpoints and adverse events) from the same trial that cohort characteristics and treatment effects were taken, pioglitazone represents a cost-effective treatment option in the German setting. In view of these outcomes, pioglitazone represents a valid treatment option for patients with a history of macrovascular events both in clinical and economic terms. It will be of interest in the future to assess the cost-effectiveness of pioglitazone for the remaining (approximately 50%) German type 2 diabetes patients without macrovascular disease who are likely to benefit from improved glycaemic control and the potential stabilization of pancreatic cell function prior to the development of costly diabetes-related complications.

## Conclusion

Based on outcomes from PROactive this modelling study demonstrates that the addition of pioglitazone to current treatment regimens in the German setting is likely to be viewed as good value for money over patient lifetimes. There were benefits in terms of both projected life expectancy and quality-adjusted life expectancy accompanied by cost savings from complications avoided and increased treatment and overall total lifetime costs.

## Competing interests

This analysis was funded by an unrestricted grant from Takeda who are manufacturers of pioglitazone hydrochloride.

WAS was engaged in the PROactive trial as a national coordinator and in that study he lead the Düsseldorf trial site. He has received honorarium from Takeda as a member of the International Diabetes Advisory Board.

GG, KMEA and WJV are all current or former employees of IMS Health who have received consultancy fees from Takeda.

MMB has received honoraria for symposia sponsored by Takeda.

EE is the chairman of the PROactive study and has received compensation for this and honoraria for symposia sponsored by Takeda.

## Authors' contributions

WAS contributed to and critically appraised the manuscript. GG performed the modelling analyses for the study and critically appraised the manuscript. KMEA contributed to the modelling analyses and prepared the manuscript. MMB contributed to and critically appraised the manuscript. EE contributed to and critically appraised the manuscript. WJV conceived of the study, contributed to the modelling analyses and preparation of the manuscript.

## Supplementary Material

Additional file 1 **Summary of events and event rates from the PROactive study.** The table presents the annual hazard rates observed over months 0–36 of the PROactive study and incorporated into the model. Click here for file

Additional file 2 **Disutility values used in the base case and sensitivity analyses.** The table presents the quality of life utility values used within the model. Click here for file

Additional file 3 **Summary of sensitivity analysis results for pioglitazone versus placebo.** The table presents the summary results of all sensitivity analyses performed in the cost-effectiveness evaluation of pioglitazone versus placebo. Click here for file
