# Supplementary material for: Cost-effectiveness of pioglitazone in type 2 diabetes patients with a history of macrovascular disease: a German perspective
Source: Cost Eff Resour Alloc. 2009 May 5;7:9. doi: 10.1186/1478-7547-7-9 (PMC2688482; doi:10.1186/1478-7547-7-9)
Supplement: Additional file 1 — Summary of events and event rates from the PROactive study. The table presents the annual hazard rates observed over months 0–36 of the PROactive study and incorporated into the model. [file 1478-7547-7-9-S1.doc]

|  | **Annual hazard rate** | | | **Hazard ratio** | | | | **Lognormal distribution** | |
| --- | --- | --- | --- | --- | --- | --- | --- | --- | --- |
|  | **Months** | **PIO** | **PLA** | **Months** | **Estimate** | **Lower** | **Upper** | **μ** | **σ** |
| Death (all causes) | 0–36+ | 2.47% | 2.58% | 0–36+ | 0.96 | 0.78 | 1.18 | –0.0435 | 0.105 |
| CV death | 0–36+ | 1.77% | 1.89% | 0–36+ | 0.94 | 0.74 | 1.2 | –0.0618 | 0.1234 |
| MI (excluding silent MI) | 0–36 | 1.49% | 1.81% | 0–36+ | 0.81 | 0.62 | 1.06 | –0.2108 | 0.1372 |
| Silent MI | 0–36 | 0.34% | 0.36% | 0–36+ | 0.9 | 0.52 | 1.55 | –0.1101 | 0.2805 |
| ACS | 0–36 | 0.90% | 1.09% | 0–36+ | 0.78 | 0.55 | 1.11 | –0.2456 | 0.1782 |
| CABG | 0–36 | 0.93% | 1.11% | 0–36+ | 0.83 | 0.6 | 1.15 | –0.1840 | 0.1663 |
| PCI | 0–36 | 1.73% | 2.13% | 0–36+ | 0.9 | 0.69 | 1.17 | –0.1037 | 0.135 |
| Stroke | 0–36 | 1.30% | 1.65% | 0–36+ | 0.81 | 0.61 | 1.07 | –0.2138 | 0.1448 |
| Major leg amputation | 0–36 | 0.39% | 0.38% | 0–36+ | 1.01 | 0.58 | 1.73 | 0.0062 | 0.2774 |
| Bypass surgery/revascularization of leg | 0–36 | 1.63% | 1.29% | 0–36+ | 1.25 | 0.9 | 1.73 | 0.2196 | 0.167 |
| TIA | 0–36 | 0.55% | 0.60% | 0–36+ | 0.86 | 0.54 | 1.35 | –0.1563 | 0.2333 |
| Retinal photocoagulation | 0–36 | 3.79% | 3.78% | 0–36+ | 1.01 | 0.82 | 1.25 | 0.0098 | 0.1072 |
| Non-serious heart failure | 0–36 | 2.92% | 2.04% | 0–36+ | 1.5 | 1.18 | 1.91 | 0.4068 | 0.1213 |
| Hospital admission for heart failure | 0–36 | 2.92% | 2.09% | 0–36+ | 1.4 | 1.1 | 1.8 | 0.3397 | 0.1264 |
| Oedema | 0–12 | 25.38% | 11.91% | 0–12+ | 2.09 | 1.8 | 2.42 | 0.7356 | 0.0757 |
|  | 12–36 | 9.52% | 5.79% | 12–36+ | 1.46 | 1.18 | 1.8 | 0.3778 | 0.1075 |
| Hospital admissions | 0–36 | 31.98% | 35.56% | 0–36+ | 0.93 | 0.86 | 1.01 | –0.0676 | 0.0412 |
| ICU admissions (subgroup of hospital admissions) | 0–36 | 6.31% | 6.66% | 0–36+ | 0.88 | 0.76 | 1.01 | –0.1332 | 0.0749 |

ACS, acute coronary syndrome; CABG, coronary artery bypass graft; CV, cardiovascular; ICU, intensive care unit; MI, myocardial infarction; PCI, percutaneous transluminal angioplasty; PIO, pioglitazone; PLA, placebo; TIA, transient ischaemic attack.

Log-normal distributions were defined by the terms μ and σ where m and s correspond to the geometric mean [exp(μ)] and the geometric standard deviation [exp(σ)].

Table taken with permission from: Diabetic Medicine 2007 24, 982-1002.
