# Supplementary material for: Cost-effectiveness of pioglitazone in type 2 diabetes patients with a history of macrovascular disease: a German perspective
Source: Cost Eff Resour Alloc. 2009 May 5;7:9. doi: 10.1186/1478-7547-7-9 (PMC2688482; doi:10.1186/1478-7547-7-9)
Supplement: Additional file 2 — Disutility values used in the base case and sensitivity analyses. The table presents the quality of life utility values used within the model. [file 1478-7547-7-9-S2.doc]

|  | **Disutility data used in base case and sensitivity analyses** | | |
| --- | --- | --- | --- |
| **Event** | **Event utility** | **Follow-up disutility** | **Reference/comment** |
| Stroke | -0.115 | -0.115 | [31] |
| Leg amputation (major, above ankle) | -0.272 | -0.272 | [31] |
| Diabetes intervention insulin | -0.049 | 0 | [31] |
| BMI (for each unit BMI>25kg/m^2^) | -0.0061 | 0 | [31] |
| MI (excluding silent MI) | -0.129 | -0.078 | [35] |
| Acute coronary syndrome (ACS) | -0.129 | -0.078 | [35]Assumed comparable with MI |
| CABG only | -0.059 | -0.059 | [36] |
| PCI only | -0.042 | -0.042 | [36] |
| Bypass surgery/revascularization of leg | -0.059 | 0 | [36] Assumed comparable to CABG |
| Transient ischemic attack (TIA) | -0.044 | 0 | [36] |
| Hospitalization for CHF | -0.121 | -0.181 | [35] |
| Non-serious heart failure | -0.0605 | 0 | Assumed half UKPDS disutility for serious heart failure |
| Oedema | -0.01 | 0 | Assumed 1% decrement in quality of life |

Utilities for events not given specific for the PROactive model are defined in Palmer *et al.* 2004 [19].
