# Supplementary material for: Cost-effectiveness of pioglitazone in type 2 diabetes patients with a history of macrovascular disease: a German perspective
Source: Cost Eff Resour Alloc. 2009 May 5;7:9. doi: 10.1186/1478-7547-7-9 (PMC2688482; doi:10.1186/1478-7547-7-9)
Supplement: Additional file 3 — Summary of sensitivity analysis results for pioglitazone versus placebo. The table presents the summary results of all sensitivity analyses performed in the cost-effectiveness evaluation of pioglitazone versus placebo. [file 1478-7547-7-9-S3.doc]

Summary of sensitivity analysis results for pioglitazone versus placebo

| **Sensitivity analysis** | **Quality-adjusted life expectancy (QALYs)** | | | **Lifetime direct costs (€)** | | | **ICER (€ per QALY gained)** |
| --- | --- | --- | --- | --- | --- | --- | --- |
|  | **Pioglitazone** | **Placebo** | **Difference** | **Pioglitazone** | **Placebo** | **Difference** |  |
| *Base case* | *7.543 (0.102)* | *7.422 (0.102)* | *0.120* | *105,433 (2,650)* | *103,834 (2,618)* | *1,599* | *13,294* |
| Beta-cell improvement | 7.554  (0.102) | 7.422 (0.102) | 0.132 | 102,954 (2,651) | 103,834 (2,618) | -880 | Pioglitazone dominant |
| Hypoglycaemic event cost of €2,555 | 7.543  (0.102) | 7.422 (0.102) | 0.120 | 105,623 (2,652) | 103,941 (2,618) | 1,681 | 13,980 |
| 10 year time horizon | 5.256 (0.053) | 5.231 (0.051) | 0.025 | 54,718 (1,157) | 53,990 (1,126) | 728 | 29,081 |
| 20 year time horizon | 7.092 (0.087) | 7.021 (0.088) | 0.071 | 89,521 (2,070) | 88,804 (2,054) | 718 | 10,144 |
| Same HbA1c with PIO and PLA | 7.541 (0.106) | 7.422 (0.102) | 0.119 | 105,246 (2,651) | 103,834 (2,618) | 1,412 | 11,870 |
| No risk adjustment for age | 7.574 (0.106) | 7.458 (0.100) | 0.117 | 98,400 (2,525) | 96,098 (2,384) | 2,302 | 19,692 |
| 0% discount rates | 11.947 (0.209) | 11.666 (0.205) | 0.281 | 199,267 (6,279) | 195,577 (6,052 | 3,690 | 13,123 |
| 10% discount rates | 5.339 (0.060) | 5.280 (0.060) | 0.060 | 65,732 (1,427) | 64,734 (1,449) | 998 | 16,717 |
| CORE QoL estimation method, using CODE‑2 CVD disutilities | 6.507 (0.091) | 6.390 (0.092) | 0.117 | 105,433 (2,650) | 103,834 (2,618) | 1,599 | 13,660 |
| Oedema disutility included | 6.497 (0.091) | 6.383 (0.092) | 0.113 | 105,433 (2,650) | 103,834 (2,618) | 1,599 | 14,118 |
| Hospitalization for heart failure disutility | 6.382 (0.089) | 6.302 (0.090) | 0.079 | 105,433 (2,650) | 103,834 (2,618) | 1,599 | 20,113 |
| Non-serious heart failure disutility | 6.482 (0.091) | 6.373 (0.091) | 0.108 | 105,433 (2,650) | 103,834 (2,618) | 1,599 | 14,757 |
| "Worst case" disutilities included | 6.334 (0.088) | 6.270 (0.090) | 0.064 | 105,433 (2,650) | 103,834 (2,618) | 1,599 | 24,807 |
| "All" CVD disutilities included | 6.264 (0.087) | 6.186 (0.088) | 0.078 | 105,433 (2,650) | 103,834 (2,618) | 1,599 | 20,623 |
| Cost pioglitazone treatment +20% | 7.543  (0.102) | 7.422 (0.102) | 0.120 | 106,981 (2,663) | 103,834 (2,618) | 3,147 | 26,162 |
| Cost pioglitazone treatment -20% | 7.543  (0.102) | 7.422 (0.102) | 0.120 | 103,885 (2,637) | 103,824 (2,618) | 51 | 424 |
| Complication and management costs +20% | 7.543  (0.102) | 7.422 (0.102) | 0.120 | 111,788 (2,398) | 110,038 (2,896) | 1,750 | 14,553 |
| Complication and management costs ‑20% | 7.543  (0.102) | 7.422 (0.102) | 0.120 | 99,078 (2,378) | 97,631 (2,354) | 1,447 | 12,035 |

Values shown are means with standard deviation in parentheses. ICER = incremental cost-effectiveness ratio; QALY = quality-adjusted life years; QoL = quality of life; CODE‑2 = cost of diabetes in Europe-type 2; CVD = cardiovascular disease.
